# Myocardial infarction showing non-ischaemic late gadolinium enhancement pattern in the mid-septum: a case report

**DOI:** 10.1093/ehjcr/ytae535

**Published:** 2024-09-25

**Authors:** Luigi Tassetti, Ludovica Massa, Gianluca Pontone

**Affiliations:** Department of Perioperative Cardiology and Cardiovascular Imaging, Centro Cardiologico Monzino IRCCS, via Carlo Parea 4, 20138 Milan, Italy; Department of Biomedical Sciences and Community Health, University of Milan, via Mangiagalli 31, 20133 Milan, Italy; Department of Perioperative Cardiology and Cardiovascular Imaging, Centro Cardiologico Monzino IRCCS, via Carlo Parea 4, 20138 Milan, Italy; Department of Biomedical, Surgical and Dental Sciences, University of Milan, via della Commenda 10, 20122 Milan, Italy

**Keywords:** Cardiac magnetic resonance, Non-ischaemic late gadolinium enhancement, Myocardial infarction, Septal vascularization, Case report

## Abstract

**Background:**

Cardiac magnetic resonance (CMR) is gaining an important role in the setting of acute coronary syndromes: it gives prognostic information based on oedema and late gadolinium enhancement (LGE) extension in acute myocardial infarction, and has a diagnostic value in myocardial infarction with nonobstructive coronary arteries (MINOCA) thanks to its capability to distinguish, based on different LGE patterns, ischaemic and non-ischaemic myocardial injuries.

**Case summary:**

We describe a case of acute myocardial infarction involving a recurrent apical branch showing an atypical intramyocardial LGE pattern in the medium inferior septum.

**Discussion:**

An intramyocardial LGE pattern might be determined by an ischaemic injury involving the interventricular septum. The knowledge of this misleading LGE pattern is important to adequately interpret CMR findings in this clinical setting.

**Learning points**

- In acute myocardial infarction involving septal vascularization, an atypical late gadolinium enhancement and oedema pattern, apparently ‘non-ischaemic’, can be identified with cardiac magnetic resonance; different pathophysiological hypothesis might explain these findings.

## Introduction

Cardiac magnetic resonance (CMR) is a diagnostic imaging technique acquiring progressive importance in many different settings, including acute coronary syndromes (ACSs).^1^ It is well known its role in the myocardial infarction with ST-segment elevation (STEMI) for prognostic stratification, providing information about oedema extension and extent of late gadolinium enhancement (LGE).^2^ Cardiac magnetic resonance is also crucial in the diagnostic workup of myocardial infarction with nonobstructive coronary arteries (MINOCA) in order to exclude other possible non-ischaemic cardiac causes of myocardial injury such as myocarditis.^3^ Late gadolinium enhancement is the most important tool we have to differentiate ischaemic and non-ischaemic myocardial injuries, the former characterized by a subendocardial or transmural LGE pattern, the latter by a subepicardial or intramyocardial LGE pattern. Furthermore, T1 and T2 mapping are gaining importance in tissue characterization in the aforementioned settings.^4^

We present a case of a 65-year-old man with a diagnosis of inferolateral STEMI who underwent primary percutaneous coronary intervention (PCI) on a left anterior descending artery (LAD) with a recurrent apical branch. Cardiac magnetic resonance was performed in order to assess the extension of myocardial oedema and LGE. This case report provides evidence of the growing importance of CMR in the acute ischaemic setting and highlights the possibility that an intramyocardial LGE pattern in the interventricular septum, as already previously reported,^5^ may be observed in the context of ischaemic heart disease.

## Summary figure

**Figure:**
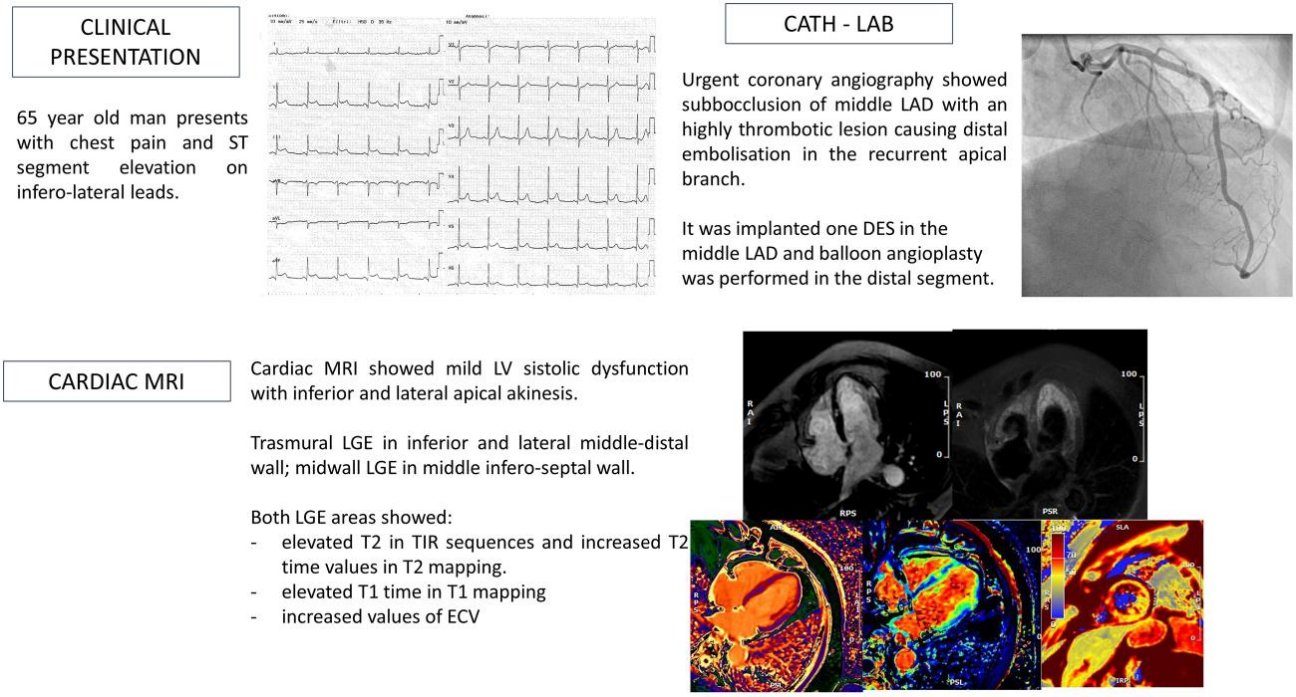


## Case presentation

**Figure 1:**
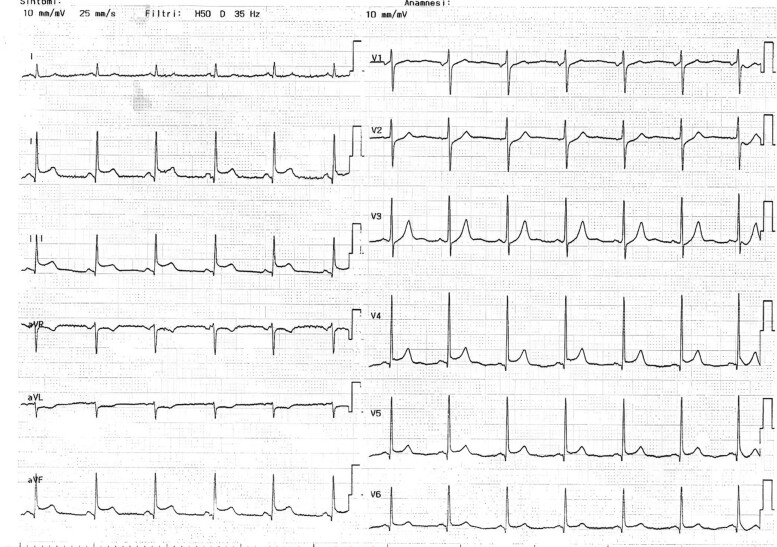
ECG at clinical presentation showing a pattern consistent with inferolateral STEMI.

**Figure 2:**
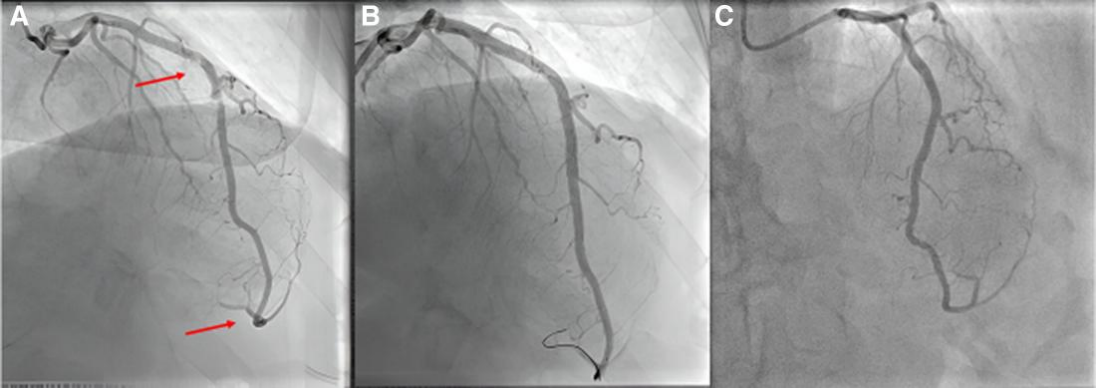
From left to right: (*A*) coronary angiography showing subtotal occlusion of the middle segment of LAD (upper arrow) and evidence of distal embolization with thrombotic apposition in the recurrent apical branch (lower arrow); (*B* and *C*) coronary angiography showing complete vessel recanalization after PCI.

A 65-year-old man with a medical history of dyslipidaemia and psoriatic arthritis was admitted to our emergency department for angina and electrocardiogram (ECG) showing a pattern consistent with inferolateral STEMI (*Figure 1*). Physical examination was unremarkable. Aspirin loading dose of 250 mg i.v. was administered, and urgent coronary angiography was performed showing a subtotal occlusion of the middle segment of LAD associated with significant thrombus burden. The distal segment of LAD, ending with a recurrent apical branch extending to the inferior and inferior-septal medium-distal wall, showed a thrombotic apposition presumably due to distal embolization of the thrombotic mass of the middle segment. A drug-eluting stent was implanted in the mid-LAD and balloon angioplasty performed to the distal LAD (*Figure 2*). During PCI, 100 IU/kg of unfractionated heparin and prasugrel loading dose of 60 mg were administered.

**Figure 3:**
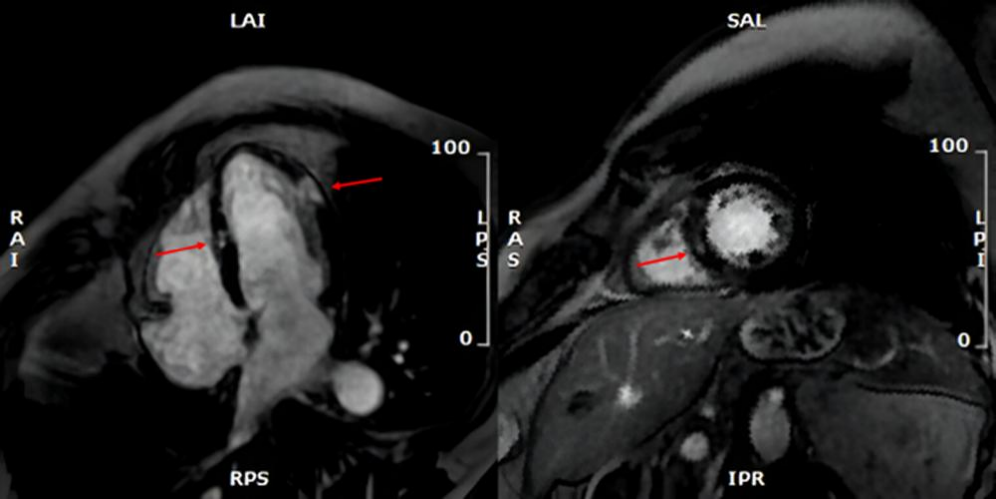
From left to right: (*A*) LGE horizontal long axis and (*B*) medium short axis sequences, with arrows pointing to the inferior-septal segment with intramyocardial LGE pattern and the lateral segment with a typical ischaemic transmural pattern.

**Figure 4:**
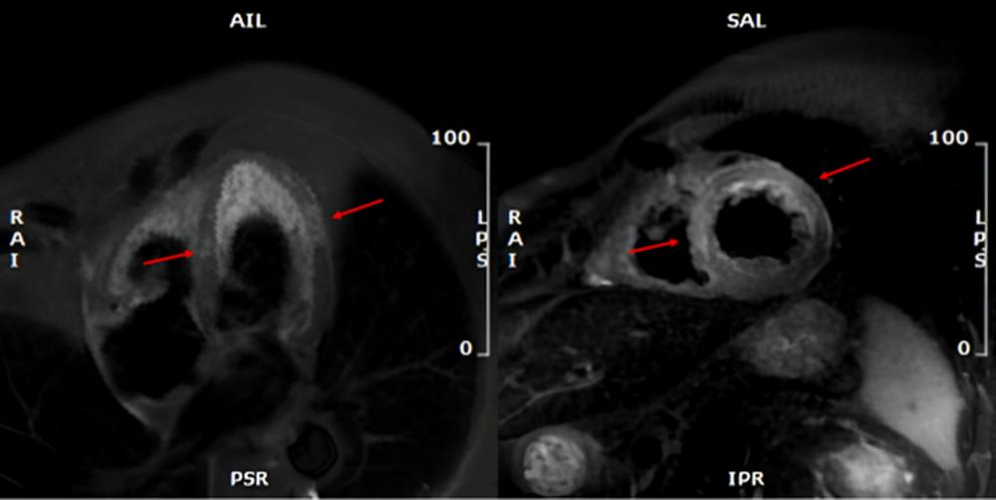
From left to right: (*A*) horizontal long axis and (*B*) medium short axis TIR T2w sequences with arrows pointing to oedema areas with the same pattern observed for LGE.

**Figure 5:**
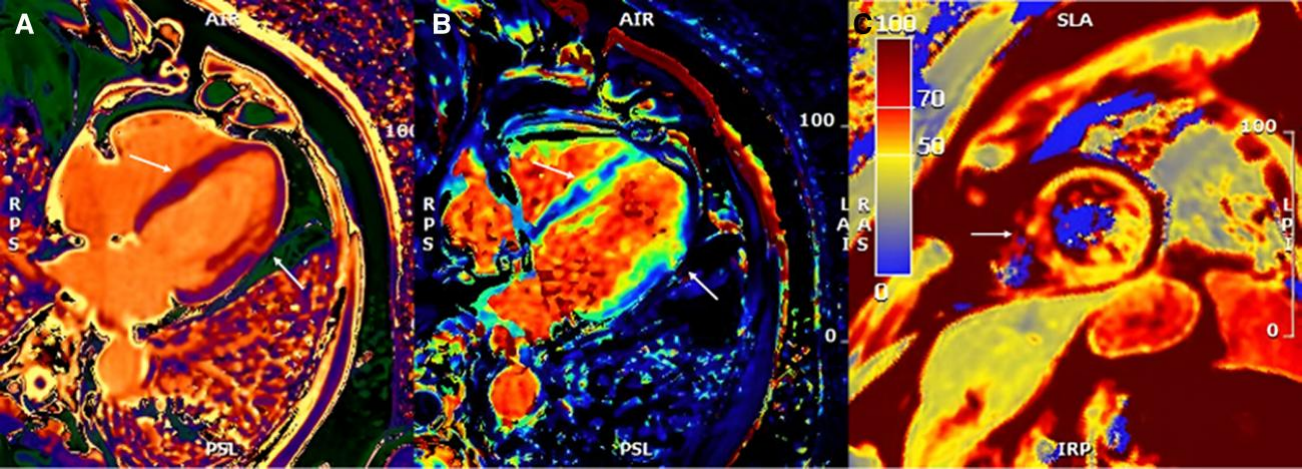
From left to right: (*A*) T1 mapping and (*B*) extracellular volume map, in horizontal long axis view, with arrows pointing to areas with incremented values of T1 and extracellular volume, the inferior-septal segment with intramyocardial pattern and the lateral segment with typical ischaemic transmural pattern, respectively; (*C*) T2 mapping medium short axis view with arrow pointing to the medium inferior septum areas with incremented T2 values.

Cardiac magnetic resonance, on 1.5 T system (GE Medical System Discovery MR450), was performed 6 days after PCI, showing mild systolic LV dysfunction with inferior and lateral apical akinesis; LGE with an ischaemic transmural pattern (>75% of wall thickness) was identified in the inferior and lateral medium-distal wall; in the mid-segment of the inferior-septal wall, an area of LGE with intramyocardial pattern typically considered non-ischaemic was noted (*Figure 3*). Both areas of LGE showed a concomitant increased transversal relaxation time signal in triple inversion recovery (TIR) T2 weighted (T2w) sequences and increased T2 time values in the T2 mapping sequences [average T2 70 ms, normal value (n.v.) < 50 ms], consistent with acute oedema and showing the same pattern as well as extending in the same area of the LGE distribution (*Figure 4*). Moreover, T1 time in T1 mapping sequences and extracellular volume (ECV) showed increased values [average T1 1200 ms (n.v. 950–1050 ms) and average ECV 50 ms (25–33 ms), respectively] in the same regions and with the same pattern of the aforementioned LGE areas, the inferior-septal segment with intramyocardial pattern and the lateral segment with typical ischaemic transmural pattern, respectively (*Figure 5*). These findings were characteristics for an acute myocardial injury, and the localization in the medium inferior-septal segment was consistent with the distribution territory of the recurrent LAD involved in myocardial infarction.

The patient was discharged on dual antiplatelet therapy (DAPT) consisting of aspirin 100 mg and prasugrel 10 mg once a day, with the recommendation of DAPT for one year and, after this period, to continue lifelong therapy with aspirin only, in accordance with current ACS guidelines.^1^ At six-month follow-up, the patient is in stable condition, reporting no symptoms.

## Discussion

We present a case of an inferolateral acute myocardial infarction of a LAD with a recurrent apical branch with the coexistence of transmural LGE in the inferior and lateral apical segments (ischaemic pattern) and an intramyocardial LGE in the inferior medium septum, with an intramyocardial pattern normally related to non-ischaemic injuries, such as myocarditis, sarcoidosis, or certain cardiomyopathies.^6^

The segment presenting with intramyocardial LGE showed findings consistent with acute oedema (T2 elevation on mapping and T2w sequences) with a similar distribution of LGE, therefore implying the presence of an ongoing injury^7^ in a segment consistent with the territory distribution of the recurrent LAD involved in the infarction.

Finally, the distal embolization of the atherothrombotic lesion from the middle segment of the LAD to the recurrent apical branch, clearly evident at the coronary angiography and probably holding part to the septum vascularization, was the most reasonable mechanism called in cause in the explanation of the mid-septal lesion detected by CMR.

This misleading LGE pattern was previously described by Iwakami *et al*.^5^ in a case report of a MINOCA, with a positive ergonovine test showing vasospasm of the septal branch that supplied the territory where the intramyocardial LGE was identified; that finding was further supported by endomyocardial biopsy. A previous study in patients with hypertrophic cardiomyopathy (HCM) going through percutaneous transluminal septal myocardial ablation (PTSMA), determining an ethanol-induced infarction through septal branches, also showed similar LGE patterns.^8^ To date, the evidence about this topic is limited to these case reports or derived from other setting (e.g. PTSMA in HCM) and there is a need for comparative human histological data.

An ischaemic lesion determining a septal intramyocardial LGE pattern held a pathophysiological foundation into prior anatomical studies showing an heterogeneous vascularization of the septum. The interventricular septum is vascularized by septal branches, which can vary in the number, size, and distribution and are four on average.^9^ In addition, it is debated whether the right ventricular side of the septum is partially perfused directly from the right ventricular cavity, through the thebesian veins.^10^

For this peculiar vascularization, LV endocardium might be spared because of the interventricular complementary work of multiple branches.

## Conclusion

The evidence of an intramyocardial typically ‘non-ischaemic’ LGE pattern in the interventricular septum should not be a finding of exclusion for ischaemic heart disease, which has always to be put in differential diagnosis if the clinical context is consistent.

We suggest an integrated approach, which takes into consideration of CMR data together with clinical and familiar history and coronary angiography, in order to elaborate a correct diagnosis and not be misled by the LGE pattern.

## Supplementary Material

ytae535_Supplementary_Data

## Data Availability

All available data are presented within the manuscript.

## Lead author biography

Doctor Luigi Tassetti is a cardiologist currently working at IRCCS Monzino Cardiological Hospital (Milan, Italy), in the Cardiovascular Imaging Department from 2022. Main field of interests are cardiac magnetic resonance, cardiac CT, and echocardiography. Before working at Monzino Hospital, he attended fellowships in Cardiac Magnetic Resonance at San Donato Hospital (Milan, Italy) and UZ Leuven Hospital (Belgium), and worked in Cardiomyopathy Unit in Florence (director Prof. Olivotto) from 2019 to 2021.

## Notes

**Consent:** The authors confirm that written consent for submission and publication of this case report including images and associated text has been obtained from the patient in line with COPE guidance.

**Funding:** This research received no external fundings.
